# Comprehending the three-dimensional mandibular morphology of facial asymmetry patients with mandibular prognathism

**DOI:** 10.1186/s40510-017-0197-6

**Published:** 2017-12-15

**Authors:** Hideki Kamata, Norihisa Higashihori, Hiroki Fukuoka, Momotoshi Shiga, Tatsuo Kawamoto, Keiji Moriyama

**Affiliations:** 1 Section of Maxillofacial Orthognathics, Department of Maxillofacial/Neck Reconstruction, Graduate School, Tokyo Medical and Dental University, Tokyo, Japan; 2 Division of Orofacial Functions and Orthodontics, Department of Health Improvement, Faculty of Dentistry, Kyushu Dental University, Kitakyushu, Japan

**Keywords:** 3D, Mandibular prognathism, Facial asymmetry, Mandibular morphology

## Abstract

**Background:**

The purpose of this study was to elucidate the factors that cause facial asymmetry by comparing the characteristics of the mandibular morphology in patients with mandibular prognathism with or without facial asymmetry using three-dimensional computed tomography (3D-CT).

**Methods:**

We studied 28 mandibular prognathism patients whose menton deviated by ≥ 4 mm from the midline (FA group, *n* = 14) and those with a < 4-mm deviation (NA group, *n =* 14). DICOM data from multislice CT images were reconstructed and analysed using 3D image analysing software. Mandibular structures were assessed via linear, angular, or volumetric measurements and analysed statistically.

**Results:**

The lengths of the ramal and body components and condylar volume in the FA group were significantly greater on the nondeviated side than those on the deviated side. The mandibular body length of the nondeviated side in the FA group was significantly longer than that of the NA group. Other components of the FA group did not significantly differ from those of the NA group.

**Conclusions:**

Imbalances in the sizes of the ramal and body components as well as the increased body length of the nondeviated side in the FA group compared with that of the NA group may contribute to facial asymmetry in patients with mandibular prognathism.

## Background

Completely symmetrical faces are almost nonexistent. Most people have some degree of asymmetry in their facial structures [1, 2]. Using triangulation, Sharad and Joshi assessed the asymmetry of the component areas of the facial complex in college students with normal occlusion and no history of orthodontic therapy, and found that one side of the facial surface area was significantly larger than that of the other [3]. Furthermore, a study on craniofacial morphology using cone-beam CT showed minor asymmetry in 30 orthodontic patients [4]. Therefore, it can be concluded that facial asymmetry is a common phenomenon in the normal human craniofacial complex. However, there are wide variations in the facial asymmetry of patients who need orthodontic treatment—from cases in which occlusion can be improved by orthodontics alone to cases of severe asymmetry where orthognathic surgery is needed to improve occlusion and/or facial features. Regardless of the issue resulting in facial asymmetry, investigating the characteristics and causes of asymmetry is important in orthodontic diagnosis and treatment.

Many years of study have been dedicated to elucidating the factors that cause facial asymmetry. These studies have been based on the use of conventional two-dimensional (2D) images such as frontal facial photographs or cephalometric radiographs. However, it is difficult to conduct evaluations using conventional 2D analysis because of distortion and magnification problems, especially in cases of asymmetry [5]. Recent developments in 3D measuring devices and image analysis software have resolved these problems. Among these measuring devices, 3D computed tomography (3D-CT) has enabled us to obtain 3D hard- and soft-tissue data at the same time with fewer magnification and distortion problems [6, 7]. The 3D measurements of the length and angle of the midfacial, mandibular, and cranial bases by Baek et al. showed that menton deviation was significantly correlated with ramal height differences between the two sides, leading the authors to conclude that greater growth of the ramus was one of the causes of asymmetry in skeletal class III patients [8]. Furthermore, You et al. showed that ramal height (condylion superior–gonion midpoint) and menton deviation were strongly correlated, implying differences in deviated and nondeviated ramal heights. However, they did not mention whether asymmetry was caused by one-sided hyper- or hypo-active mandibular growth [9].

The present study was conducted to elucidate the factors that cause facial asymmetry by comparing the characteristics of mandibular morphology in patients with mandibular prognathism with or without facial asymmetry using 3D-CT.

## Methods

Twenty-eight patients with mandibular prognathism (ANB < 0°) who had undergone orthognathic surgical procedures at the Tokyo Medical and Dental University Dental Hospital were included in this study. On posteroanterior cephalometric radiographs taken at the initial examination, the midline was defined as the line that passed through the crista galli of the ethmoid bone, perpendicular to the Lo-Lo′ line. Patients whose menton deviated 4 mm or more (mean 8.9 ± 4.3 mm) from the midline were assigned to the group considered to have skeletal mandibular prognathism with facial asymmetry (FA group: 14 cases). Patients who had less than 4 mm deviation (mean 1.6 ± 0.8 mm) were assigned to the group with skeletal mandibular prognathism without facial asymmetry (NA group: 14 cases). In both groups, we defined the deviated side as the one with menton deviation. This classification was based on the report of Baek et al. [8]. Patients with congenital disorders, such as cleft lip and/or cleft palate, were not included. The institutional ethics committee of Tokyo Medical and Dental University approved the research protocol (approval #731).

3D-CT data were obtained from multislice CT images (SOMATOM Plus-S®; Siemens Japan, Tokyo, Japan) acquired immediately before surgery. The CT imaging conditions were as follows: 120 kV, 200 A, 15–20-s exposure time, and 4 mm/s table speed. Digital Imaging and Communications in Medicine (DICOM) data from multislice CT images were reconstructed and analysed using SimPlant OMS® software (Materialise Dental Japan, Tokyo, Japan). The data were created with 0.5-mm slice thicknesses. The segmentation level and segmentation width were 1285.5 and 1785.5 HU, respectively.

**Fig. 1:**
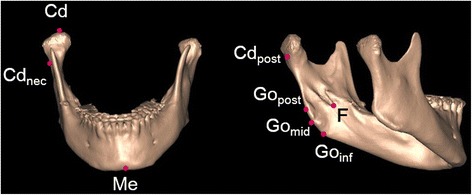
Landmarks used in this study. See Table 1 for definitions of the abbreviations

Landmarks were inscribed on the 3D model as described in Table 1 and Fig. 1.

**Table 1:** Landmarks of mandibular structures

Landmarks	Definition
Cd	Most superior point of the condylar head
Cd_nec_	Most depressed point of the lateral neck of mandible
Cd_post_	Most posterior point of the mandibular condylar base plane come in contact with the ramus
Me	Most inferior point in the symphysis
F	Most inferior point on the fossa of the mandibular foramen
Go_inf_	Most inferior point on the mandibular angle
Go_post_	Most posterior point on the mandibular angle
Go_mid_	Midpoint between Go_post_ and Go_inf_ on the mandibular angle

### Definition of the mandibular condyle

**Fig. 2:**
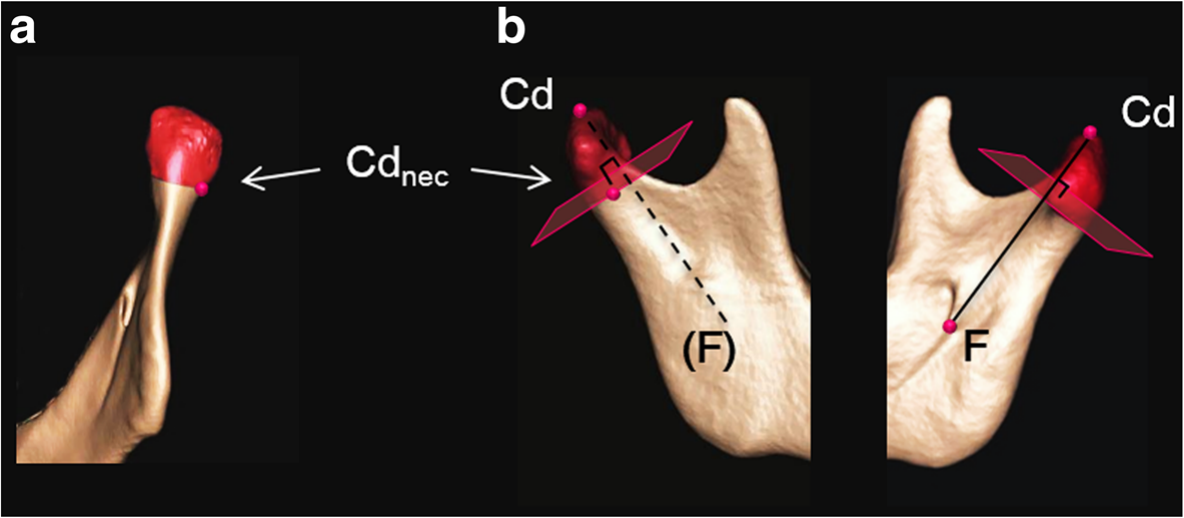
A plane was established that perpendicularly intersected with the Cd–F line and passed through Cd_neck_. The condyle was defined as the structure superior to this plane (red area). **a** Posterior view of ramus. **b** Exterior view (left) and interior view (right) of ramus

The most inferior point of the mandibular foramen (F) is recognised as a good reference point among the landmarks in the area surrounding the mandibular foramen [10]. Connecting F and the most superior point of the condyloid process (Cd) creates a reproducible axis for the mandibular ramus. We hypothesised that using the plane passing through the most recessed area of the lateral mandibular neck (Cd_neck_) orthogonal to the mandibular ramal axis (Cd–F) would be as reproducible as the plane partitioning the mandibular condyle from the rest of the ramus. Therefore, a plane was defined orthogonal to the line connecting Cd and F that passed through Cd_neck_ (hereafter referred to as the mandibular condylar base). The area superior to this plane was defined as the mandibular condyle (Fig. 2). The software measured the volume of the mandibular condyle in an automated manner.

### Linear measurements

**Fig. 3:**
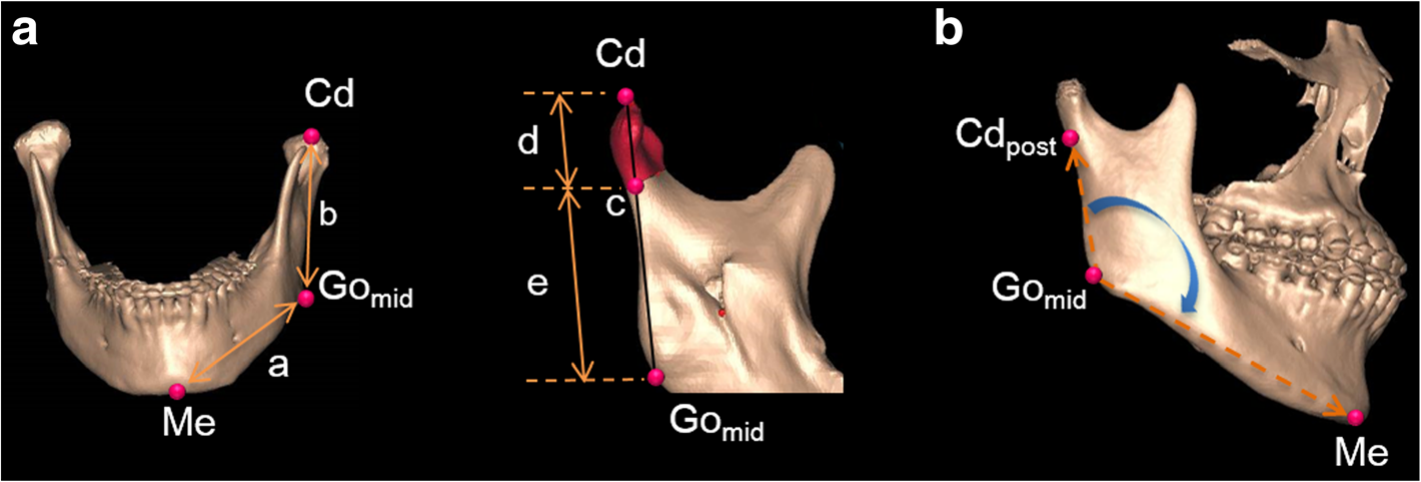
Measurements used in this study. **a** a, body length; b, ramal height; c, point of contact of the Cd–Go_mid_ line and the condylar basal plane; d, condylar height; e, inferior ramal height. **b** Gonial angle: Cd_post_–Go_mid_–Me

Body length was measured as the distance from Go_mid_ to Me. Ramal height was measured as the distance from Cd to Go_mid_ (Fig. 3a). A point was designated where the Cd–Go_mid_ line intersected with the mandibular condylar base. Condylar height was measured as the distance superior to this point along the length of the ramus. Inferior to this point was the inferior ramal height.

### Angular measurements

The gonial (Go) angle was created by the Cd_post_–Go_mid_ line and the Go_mid_–Me line (Fig. 3b).

### Condylar volume measurements and method accuracy

To examine the inter- and intra-examiner reliability of the measurements, mandibular condyles were extracted from the CT data of randomly selected cases and their volumes were measured according to the method of Kwon et al. [11]. Three orthodontists performed the volume measurements, with each one separately conducting two measurements. Measurement errors were evaluated with Dahlberg’s formula: Se = *√ΣD* ^2^ */*2*n*, where *D* is the difference between the two measurements, and *n* is the number of times the measurements were repeated. No significant inter- or intra-observer differences were observed.

### Statistical analysis

The Mann-Whitney *U* test was used to compare differences in the nondeviated and deviated sides between the two groups. The measurements on both sides of the FA group were compared with those of the NA group using the Mann-Whitney *U* test. The Wilcoxon signed-rank test was used to compare the mandibular measurements of the deviated and nondeviated sides in the FA and NA groups. Spearman’s rank correlation coefficient was used to examine correlations between the mandibular measurements.

## Results

### Comparison of the bilateral differences in the mandibular components between the FA and NA groups

To identify the mandibular components that create facial asymmetry, we compared the differences in the nondeviated and deviated sides between the FA and NA groups. Compared with the NA group, significant differences were observed in body length (*P* = 0.001), ramal height (*P* = 0.027), condylar height (*P* < 0.001), and condylar volume (*P* < 0.001) in the FA group—indicating that asymmetry was caused by the mandibular morphology (Table 2).

**Table 2:** Comparison of the bilateral differences in the mandibular components between the FA and NA groups

	FA group		NA group		
	Mean	SD	Mean	SD	*P* value
Body length difference	4.9	2.7	0.9	2.8	0.001**
Ramal height difference	4.3	5.5	0.2	3.8	0.027*
Condylar height difference	2.3	1.6	− 0.4	2.0	0.000**
Inferior ramal height difference	2.0	4.3	0.7	3.9	0.603
Condylar volume difference	530.2	261.8	− 63.5	193.7	0.000**
Gonial angle difference	1.9	1.7	− 0.5	6.7	0.125

*P < 0.05, **P < 0.01

### Bilateral comparison of the mandibular components in the FA and NA groups

In the FA group, linear measurements of body length, ramal height, and condylar height were significantly smaller on the deviated side. Condylar volume and the Go angle were also smaller on the deviated side in the FA group. There were no significant differences between the measurements on both sides in the NA group (Table 3).

**Table 3:** Bilateral comparison of the mandibular components in the FA and NA groups

	FA group					NA group				
	Deviated side		Nondeviated side			Deviated side		Nondeviated side		
	Mean	SD	Mean	SD	*P* value	Mean	SD	Mean	SD	*P* value
Body length (mm)	89.1	5.4	94.0	4.2	0.001**	87.7	5.6	88.6	6.5	0.297
Ramal height (mm)	58.6	8.4	63.0	6.0	0.008**	61.9	5.0	62.1	4.7	0.515
Condylar height (mm)	13.9	2.7	16.2	2.2	0.002**	15.5	2.3	15.1	1.5	0.611
Inferior ramal height (mm)	44.8	6.4	46.8	5.1	0.140	46.4	4.7	47.1	4.8	0.326
Condylar volume (mm^3^)	1413.1	614.1	1943.2	708.2	0.001**	1693.5	443.1	1629.7	403.0	0.056
Gonial angle (°)	125.2	5.4	127.1	5.4	0.003**	131.0	13.7	130.5	13.2	1.669

**P < 0.01

### Comparison of the mandibular components of each side in the FA group and of those in the NA group

To identify whether the linear measurements and the volume of each side of the FA group were similar to or different from those of the NA group, we compared the mandibular components of each side in the FA group and the corresponding mandibular components in the NA group. No difference was observed in the linear measurement of body length between the deviated side of the FA group and that of the NA group. In the nondeviated side of the FA group, however, body length was significantly longer than that in the NA group (*P* = 0.011) (Table 4).

**Table 4:** Comparison of the mandibular components of each side in the FA group and of those in the NA group

	FA group deviated side vs NA group	FA group nondeviated side vs NA group
Body length	*P* = 0.482	*P* = 0.011*
Ramal height	*P* = 0.246	*P* = 0.667
Condylar height	*P* = 0.227	*P* = 0.164
Inferior ramal height	*P* = 0.352	*P* = 0.769
Condylar volume	*P* = 0.227	*P* = 0.427
Gonial angle	*P* = 0.246	*P* = 0.839

*The nondeviated side of the FA group was significantly larger (*P* < 0.05) than the NA group

### Correlations between the measurements of mandibular morphology in the FA group

Many previous reports have used differences in the deviated–nondeviated sides of actual measurements to compare groups or investigate correlations [8, 9]. In this study, we used the ratios of the deviated–nondeviated sides to eliminate the effect of size differences among the mandibles and to make the evaluations more objective. Menton deviation was negatively correlated with the body length ratio (*P* = 0.044), ramal height ratio (*P* = 0.016), condylar height ratio (*P* = 0.023), inferior ramal height ratio (*P* = 0.016), and condylar volume ratio (*P* = 0.010). For the ramal height ratio, positive correlations were found with the condylar height ratio (*P* = 0.001), inferior ramal height ratio (*P* < 0.001), and condylar volume ratio (*P* = 0.044). For the condylar height ratio, a positive correlation was found with the inferior ramal height ratio (*P* = 0.003). For the inferior ramal height ratio, a positive correlation was found with the condylar volume ratio (*P* = 0.037) (Table 5).

**Table 5:** Correlations between the measurements of mandibular morphology in the FA group

Correlation coefficient (*P* value)	Body length ratio	Ramal height ratio	Condylar height ratio	Inferior ramal height ratio	Condylar volume ratio	Go angle ratio
Menton deviation	− 0.545 (0.044*)	− 0.627 (0.016*)	− 0.600 (0.023*)	− 0.629 (0.016*)	− 0.660 (0.010*)	− 0.501 (0.068)
Body length ratio		0.372 (0.190)	0.487 (0.078)	0.365 (0.200)	0.472 (0.088)	0.490 (0.076)
Ramal height ratio			0.798 (0.001**)	0.984 (0.000**)	0.544 (0.044*)	− 0.086 (0.769)
Condylar height ratio				0.732 (0.003**)	0.507 (0.064)	− 0.069 (0.814)
Inferior ramal height ratio					0.561 (0.037*)	− 0.048 (0.871)
Condylar volume ratio						0.310 (0.281)

*P < 0.05, **P < 0.01

## Discussion

The present study focused on the 3D mandibular morphology to elucidate the characteristics of facial asymmetry in Japanese jaw deformity patients with mandibular prognathism. We found that most of the components of the mandible significantly differed between the deviated and nondeviated sides in the FA group (Table 3). Furthermore, the linear measurement of the mandibular body in the nondeviated side of the FA group was larger than that of the NA group (Table 4).

Posteroanterior cephalometric analysis has been used to identify the mandibular components that cause the facial asymmetry. A 2D study by Fong et al. reported that asymmetry was found in the mandibular body in patients with menton deviation, although ramal heights were not significantly different [12]. The results were evaluated using posterior-anterior cephalometric analysis, which has limitations because of the magnification and distortion errors inherent in the projection techniques [9, 12]. To overcome these problems, 3D-CT analysis has been frequently used to evaluate factors that may induce facial asymmetry [13–17]. Our findings and those of others showed that the ramal component of the deviated side was shorter or smaller than that of the nondeviated side in patients with facial asymmetry.

The mandible is composed of a body, condyles, a chin, and several processes, including the alveolar process, coronoid process, and angular process. To identify the mandibular components that contribute to facial asymmetry, we evaluated the correlations of menton deviation with several measurements. The results showed that the ramal height ratio, condylar height ratio, condylar volume ratio, inferior ramal height ratio, and body length ratio were correlated with menton deviation. You et al. reported a similar conclusion that the ramal component and the body component both correlated with menton deviation, although they did not analyse the ramal components separately [9]. Additionally, correlations were found between the condylar component ratio (condylar volume and height ratio) and the ramal component ratio (ramal height ratio or inferior ramal height ratio), suggesting that the size of the condyle may control the ramus component.

Whether the body component is asymmetrical in terms of length remains controversial. Baek et al. showed no significant differences in body size between the two sides in patients with facial asymmetry [8]. In contrast, our results showed that body length was significantly different on the nondeviated and deviated sides (Table 3), and these findings are consistent with those of You et al. and Kwon et al. [9, 11]

The pathogenesis of facial asymmetry reportedly involves two factors: congenital and acquired factors [18]. Genetic factors and syndromic disorders are included among the congenital factors, with hemifacial microsomia as one example. Hemifacial microsomia is a disorder in which the lower half of one side of the face is underdeveloped [19]. Acquired factors, such as a functional shift during the growth period of the mandible, are known to be causes of facial asymmetry. When a functional shift is maintained for a long time, especially during the growth period, the sustained functional load on the mandible transforms the functional shift into structural asymmetry [20]. However, although imbalanced growth of the condyle induces facial asymmetry, it is difficult to distinguish whether the asymmetry has occurred through hyperactivity of the nondeviated side of the condyle or through hypoactivity of the shifted side of the condyle, or both, during the growth period. Therefore, we compared the deviated side and the nondeviated side of the FA group with those of the NA group. Our data showed significant left–right differences in the FA group (Table 3). However, we did not observe any differences between the two groups in the deviated and nondeviated sides, except for body length in the nondeviated side of the FA group, which was longer (Table 4). Based on our results, we could not conclude whether this was a result of hyperactivity in the nondeviated side or hypoactivity in the shifted side. The phenomenon of the body length of the nondeviated side of the FA group, which was larger than that of the NA group, causing the menton to move distally to the shifted side may be one of the mechanisms by which facial asymmetry occurs.

## Conclusions

We assessed mandibular morphology using 3D-CT in patients with skeletal mandibular prognathism and facial asymmetry. Imbalances in the sizes of the ramal and body components and the longer body length of the nondeviated side of the FA group compared with that of the NA group may possibly contribute to facial asymmetry in patients with mandibular prognathism.

## Abbreviations

3D-CT: Three-dimensional computed tomography
DICOM: Digital Imaging and Communications in Medicine
FA group: The group with skeletal mandibular prognathism with facial asymmetry
HU: Hounsfield unit
NA group: The group with skeletal mandibular prognathism but without facial asymmetry
